# Supplementary material for: Identification and analysis of CYP450 genes from transcriptome of Lonicera japonica and expression analysis of chlorogenic acid biosynthesis related CYP450s
Source: PeerJ. 2017 Sep 12;5:e3781. doi: 10.7717/peerj.3781 (PMC5600180; doi:10.7717/peerj.3781)
Supplement: Table S1 — Primers used for qRT-PCR [file peerj-05-3781-s001.docx]

Supplemental Table 1 Primers used for qRT-PCR

| Genes | Forward primer (5’-3’) | Reverse primer (5’-3’) | Product size (bp) |
| --- | --- | --- | --- |
| *LjPAL1* | TTTACCGACCATTTGACGC | GATGTTCGGAGGGCGTAG | 170 |
| *LjPAL2* | AGTTTACGGACCATTTGACG | GTACCGATCTTGCTTAGGCT | 158 |
| *LjPAL3* | TCTTTGGCAACGGCACAG | GTCCAGCAATGTAGGATAAGG | 225 |
| *LjC4H1* | TCTCGGGATTACCTTGGG | CTTGGCAACAATCGTGG | 136 |
| *LjC4H2* | ACACGCCGATACCTTTACT | ACCTTACCTCCCGCAAC | 205 |
| *LjC4H3* | GAAACCTTGCGTCTCCG | ATGGGCAGGGTTATTGG | 171 |
| *Lj4CL2* | GGTGATGTCGCATTCATTCTC | GAAATCTCGGACTCTGTGCTG | 122 |
| *LjC3H1* | CTATTTCGGTTCAGTAGCATTC | CGATTATTCCCTCCGTGTTC | 80 |
| *LjC3H2* | CCCACTAATGCTCCCTCACC | CCTGCTCCAAATGGCAATAAC | 210 |
| *LjHQT* | ATGAACTGTAACGGTGAAGGTG | TGAAGTGGAGGGAGGAAAC | 244 |
| *LjActin* | TGCTGGATTCTGGTGATGGT | ATTTCCCGCTCTGCTGTG | 169 |
